# The Genus *Tripleurospermum* Sch. Bip. *(Asteraceae):* A Comprehensive Review of Its Ethnobotanical Utilizations, Pharmacology, Phytochemistry, and Toxicity

**DOI:** 10.3390/life13061323

**Published:** 2023-06-05

**Authors:** Parvaneh Sheydaei, Ana Paula Duarte

**Affiliations:** 1 Health Sciences Research Centre (CICS), University of Beira Interior, 6200-506 Covilhã, Portugal; apcd@ubi.pt; 2 Faculty of Health Sciences, University of Beira Interior, 6200-506 Covilhã, Portugal

**Keywords:** *Tripleurospermum*, phytochemistry, antioxidant activity, antimicrobial activity, cytotoxicity, anti-inflammatory and analgesic effects

## Abstract

This review provides a comprehensive overview of the botany, traditional uses, phytochemistry, pharmacology, and toxicity of the genus *Tripleurospermum*. *Tripleurospermum*, a prominent genus within the family Asteraceae, is recognized for its therapeutic potential in treating various ailments, including skin, digestive, and respiratory diseases; cancer; muscular pain; and stress and as a sedative. Through extensive phytochemical studies regarding the *Tripleurospermum* species, numerous chemical compounds have been identified and classified into distinct classes, predominantly encompassing terpenes, hydrocarbons, steroids, hydrocarbons, oxygenated compounds, flavonoids, tannins, alcohols, acids, melatonin, and fragrant compounds. The findings from this review highlight the presence of bioactive compounds within the *Tripleurospermum* species that possess significant medicinal properties.

## 1. Introduction

In recent years, there has been a growing trend in the utilization of natural medicines to treat illnesses due to their reduced adverse effects, cost-effectiveness, and wide availability [1]. Although several articles have already explored the medicinal potential of active compounds, namely active molecules, derived from the genus *Tripleurospermum* in various therapeutic areas [2,3,4,5,6], it is imperative to conduct further investigations into the therapeutic and toxicological properties of this plant genus. The aim of this review was to comprehensively analyze the botanical characteristics, traditional uses, phytochemistry, pharmacology, and toxicity profiles of different *Tripleurospermum* species. Moreover, we intended to facilitate and guide future research endeavors by documenting the ethnopharmacological applications of *Tripleurospermum*. Noteworthy scientific contributions have been made on diverse facets of the genus *Tripleurospermum*, such as its pharmacology [2], toxicity [2], biochemical properties, the pharmaceutical potential of proteins and peptides derived from this genus [7,8,9,10,11,12], as well as its chemical constituents [2,13,14]. Lastly, this review provides a concise summary and outlines future research directions in the field of the *Tripleurospermum* genus. In the subsequent sections, an overview of the taxonomy, stomatal characteristics, achene, as well as the anatomical features of leaves and stems observed in various species of *Tripleurospermum* is presented. The genus *Tripleurospermum* is a member of the tribe Anthemideae, in the family Asteraceae, comprising nearly 40 species. It is closely related to the genus *Matricaria* L. which consists of approximately seven well-defined taxa [15]. In the geographical region of Turkey, *Tripleurospermum* is reported to encompass 40 species and 32 taxa [3,16,17,18,19,20,21], of which 12 exhibit distinct leaf and achene/cypsela anatomical characteristics [22]. Recent taxonomic research by Ref. [23] identified *Tripleurospermum eskilensis* as a new species, thus expanding the number of *Tripleurospermum* taxa in Turkey to 33. Notably, Turkey has been recognized as the primary source of the cultivated variety of *Tripleurospermum* [24,25]. In Russia, the genus *Tripleurospermum* comprises 17 species [26], while Iran is home to 6 species of *Tripleurospermum* [13,27].

The taxonomic classification of the genus *Tripleurospermum* remains a subject of contention, particularly due to its species’ distinct characteristics. Initially, the genus *Tripleurospermum* was classified under the genus *Matricaria* L. but was later reclassified as a separate genus based on two distinguishing factors: the unique shape of its achene and the prevalence of tetrasporic embryo sacs [4]. However, it should be noted that Refs. [5,6] have erroneously reported *Matricaria* L. as part of the *Tripleurospermum* genus. Subsequent investigations confirmed the association of *Tripleurospermum* with *Anthemis* L. rather than with *Matricaria* L. [28]. In addition, limited chromosomal information is available for both *Tripleurospermum* and *Anthemis* L. The ploidy levels observed in *Tripleurospermum* range from 2n = 2x = 18 to 2n = 3x = 27 to 2n = 4x = 36. There are four ploidy levels (2x, 3x, 4x, and 5x) in all *Tripleurospermum* species [29]. In contrast, *Matricaria L*. was found to have a single ploidy level (2x) [8]. Polyploids have been proposed as a potential mechanism contributing to the diversification and divergence of the genus *Tripleurospermum* [3,25]. Furthermore, the outermost layer of this genus’ leaves has been identified as a significant factor in taxonomic differentiation and to play a role in the development of stomatal complexes, including those found in achenes [30,31]. It is worth noting that anatomical features such as stomatal length, vascular bundle size, and palisade sclerenchyma thickness are important for distinguishing between *Tripleurospermum* species and for establishing a correlation between ploidy level and anatomical structures across different *Tripleurospermum* species [22,32].

The stomata of *Tripleurospermum* species are distributed on both the upper and lower surfaces of their leaves, with equal stomatal density reported [22]. Stomatal characteristics such as stomatal frequency, guard cell length, and stomata plastids’ diversity have been used as morphological indicators for assessing ploidy levels in different plant species [33,34,35,36]. Studies have demonstrated that polyploid plants exhibit longer stomata than diploid plants [37,38]. Furthermore, a positive correlation was observed between ploidy level, stomatal size, and altitude in *Tripleurospermum* species [3]. Taxonomically, stomatal and vascular bundle sizes are associated with the degree of polyploidy, which holds significance in distinguishing different *Tripleurospermum* species, particularly within the Turkish endemic species [30].

In all species of the genus *Tripleurospermum*, achenes exhibit a general similarity, but their anatomical structure, i.e., their pericarp, seed coat, endosperm, and seed lobe, display distinct variations among species [22], which aligns with previous findings reported by Ref. [30]. The morphology and anatomy of achenes are known to contribute to the systematic and phylogenetic understanding of the Anthemideae tribe within the Asteraceae family [15,39,40,41,42,43,44]. Furthermore, achene morphology, such as size, shape, number of ribs, slime formation, and pericarp shape, are crucial in the classification of *Tripleurospermum* taxa [31,39,43,45]. 

The leaf anatomy of *Tripleurospermum* species bears similarities to that of *Matricaria* L. [22]. The leaf anatomy of Turkish *Tripleurospermum* taxa typically consists of lower and upper epidermis, parenchymatous mesophyll, and a vascular bundle [46]. The epidermal cells are isodiametric, resulting in nearly straight walls in both the lower and upper epidermis [30]. The leaf surfaces are covered with nonglandular uniseriate and multicellular trichomes, although *T. corymbosum* has been reported to be glabrous [22,30]. The mesophyll blades primarily comprise dorsiventral and palisade parenchyma cells, similar to the mesophyll structure observed in the Asteraceae family [22,47,48]. In addition, all taxa of Tripleurospermum exhibit either one major vascular bundle or two medium-sized vascular bundles with a secondary vascular bundle [22]. It is worth noting that the large vascular bundle, which constitutes 50% of the mesophyll’s area, holds taxonomic significance in the delimitation of the taxa [22,30,47].

Regarding stem anatomy, *Tripleurospermum* stems are usually rounded, but some species, such as *T. caucasicum*, *T. melanolepis*, *T. rosellum* var. *album*, *T. parviflorum*, *T. sevanense*, *T. transcaucasicum*, and *T. monticola* [32], were reported to have slightly ridged stems with seven to nine ribs [32]. The stem anatomy of the studied *Tripleurospermum* species, including *M*. *chamomilla* [49], *T. baytopianum*, *T. caucasicum*, *T. monticola*, and *T. transcaucasicum* [50,51], generally exhibits similarities. However, the stem anatomy of *Matricaria* L. and *Tripleurospermum* is considered of minor importance for taxa delimitation [32].

## 2. Habitat, Distribution, and Ecology

The Asteraceae family represents the largest and most prominent family of plants [52]. The related extensive research has focused on the remarkable diversity of the species and genera, global distribution, and effective plant species [42].

**Figure 1:**
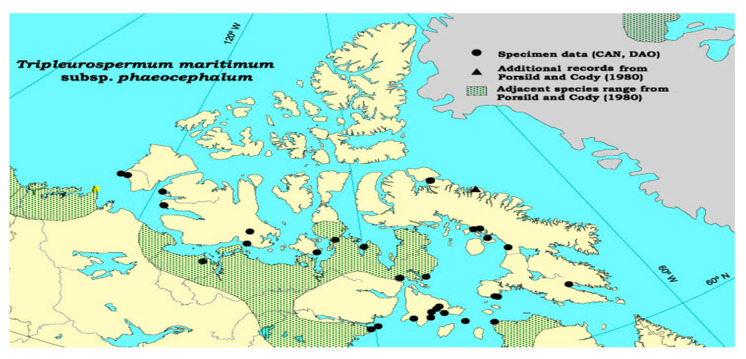
Distribution of the cytotype of *Tripleurospermum maritimum* in North America and the northern hemisphere. *●* − 2*n* = 18 [53].

*Tripleurospermum* Sch. Bip., a genus within the family, has been cultivated in various temperate regions of Europe and Asia alongside other species found in north Africa and North America [12,15,20,22,25] (Figure 1). Species belonging to this genus are characterized as herbaceous, either annual or perennial in nature [14].

## 3. Phytochemical Compounds of the Genus *Tripleurospermum*

**Figure 2:**
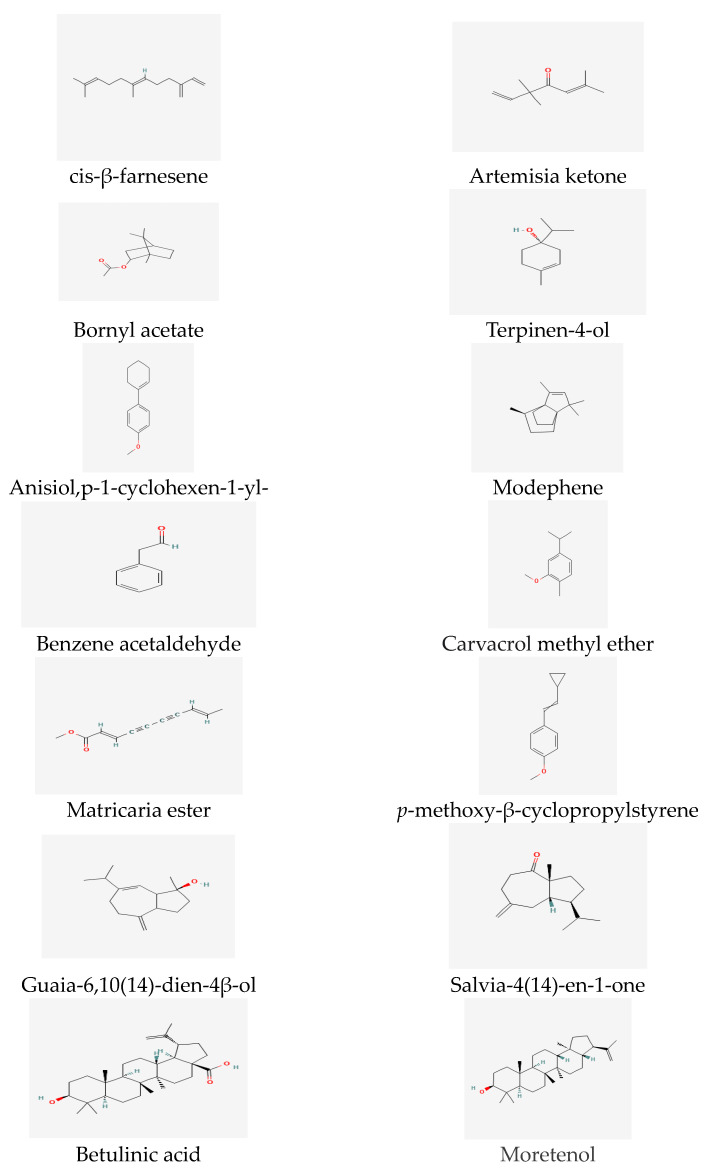

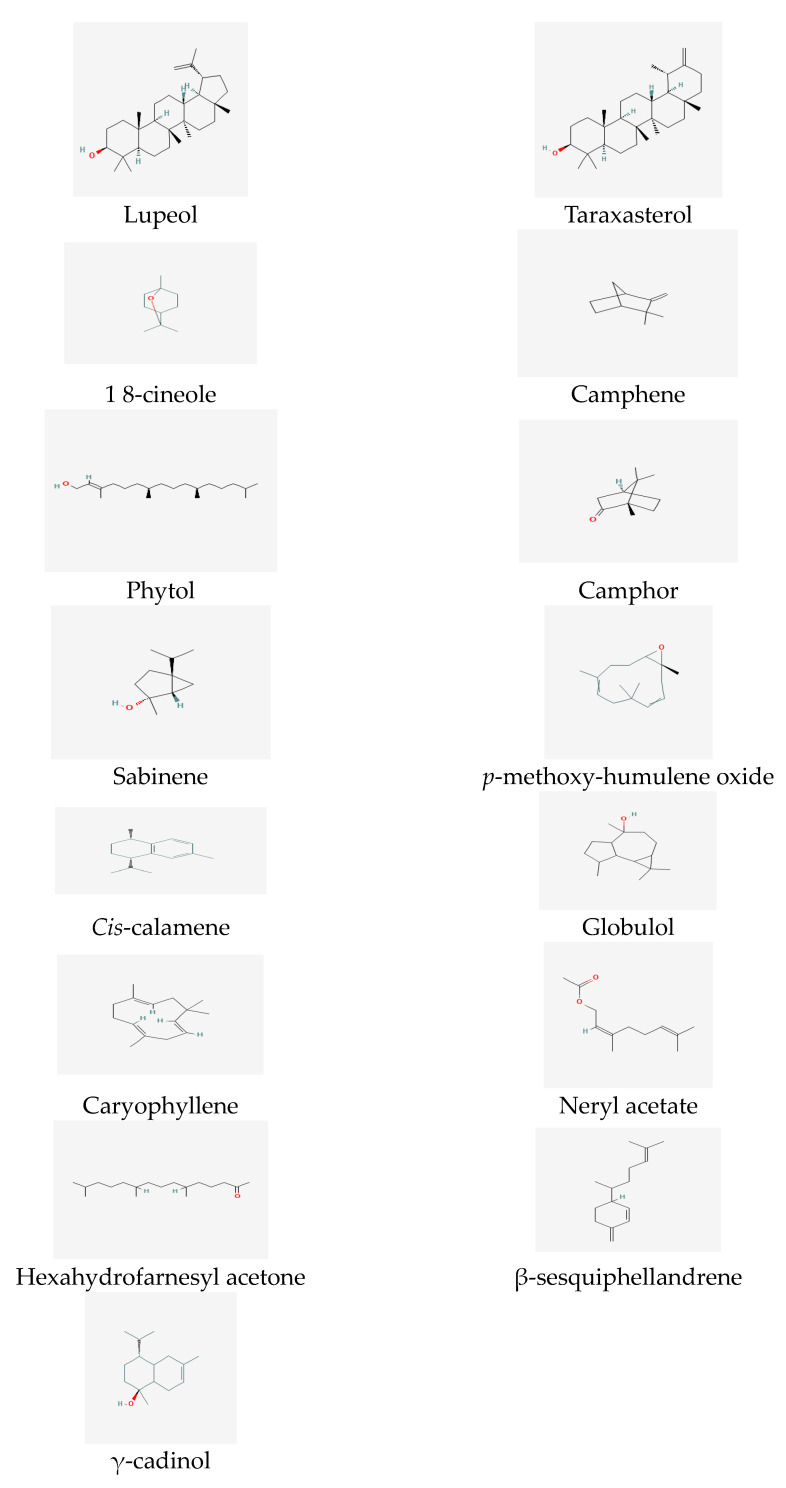
Essential oil compounds of *Tripleurospermum* spp.

The essential oil composition of *Tripleurospermum decipiens* flowers has been previously investigated, and the main reported compounds are matricaria esters [44] (Figure 2). Matricaria esters have also been identified as compounds of *T. inodorum* [54] and *T*. *disiforme* [55] (Figure 2).

The presence of terpenoids in *Tripleurospermum decipiens* was confirmed based on a reference study [56]. Furthermore, the primary compounds found in the essential oil of the aerial parts of *Tripleurospermum disiforme* include trans matricaria ester (39.93%), cis-calamenene (22.99%), (Z)-b-farnesene (12.54%), b-maaliene (7.98%) and b-sesquiphellandrene (2.22%) [57].

Previous reports have identified the significant essential oil compounds in the flowers, leaves, and stems of *T. inodorum*. In the flowers, important compounds include artemisia ketone (14.4%), terpinene-4-ol (5.5%), 1,8-cineole (5.1%), sabinene (4.7%), and tricosane (4.6%) [58] (Figure 2). The leaf is characterized by important compounds such as caryophyllene oxide (16.0%), phytol (12.1%), spathulenol (5.9%), hexahydrofarnesyl acetone (3.8%), and salvia-4(14)-en-1-one (3.5%), which exhibit relatively high percentages. The main compounds in the stem essential oil include neryl acetate (12.8%), (*E*)-β-farnesene (12.5%), phytol (12.1%), guaia-6,10(14)-dien-4β-ol (10.8%), γ-cadinol (7.8%), nonacosane (7.3%), decanoic acid (6.3%), and caryophyllene oxide (4.6%) (Figure 2).

Phytochemical research on *T. insularum* Inceer & Hayırlıoglu-Ayaz [19] has revealed that the basic compounds of the headspace and essential oils are fatty acids and n-alkanes (38.43–59.22%), including n-octacosane, linoleic acid, and n-hexacosane [59]. Additionally, sesquiterpenes (13.45%) and β-sesquiphellandrene (9.29%) were identified as the main compounds in the essential oil of the genus *T. insularum* Inceer & Hayırlıoglu-Ayaz [19]. Moreover, β-sitosterol (14.82%) and globulol (13.45%) are notable essential compounds in the headspace [59] (see Figure 2).

**Figure 3:**
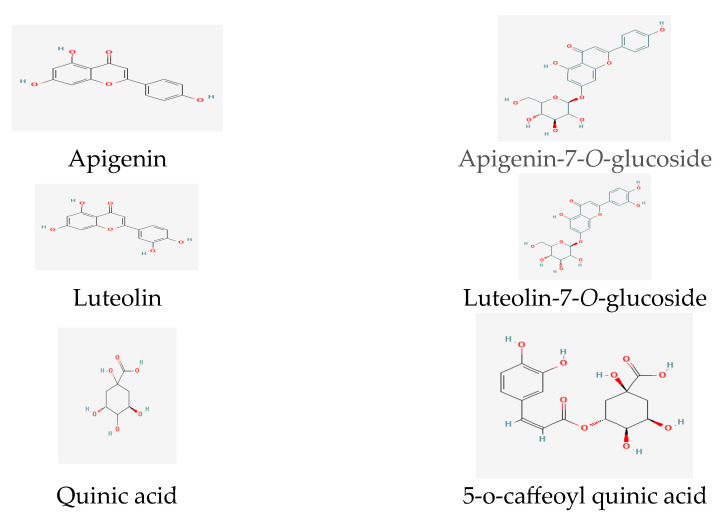
Phenolic and flavonoid compounds in *Tripleurospermum* spp.

Recent research revealed the presence of significant phenolic and flavonoid compounds in *T. inodorum*, namely apigenin, apigenin-7-*O*-glucoside, luteolin, luteolin-7-*O*-glucoside, quinic acid, and 5-*O*-caffeoyl quinic acid [60] (Figure 3).

**Figure 4:**
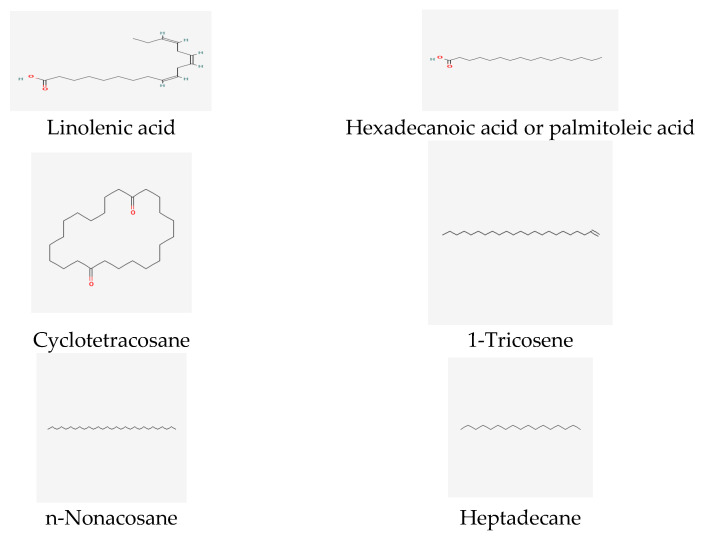
Fatty acids and hydrocarbon compounds of *Tripleurospermum* spp.

Furthermore, recent reports indicated that the genus *T. insularum* Inceer & Hayırlıoglu-Ayaz [19] contains fatty acids and n-alkanes (38.43–59.22%), such as n-octacosane, n-hexacosane, and linoleic acid (Figure 4).

Plants belonging to the genus *Tripleurospermum* encompass a wide range of phytochemicals, including terpenoids, alkanes, steroids, organic acids, and aromatic compounds [61,62]. The essential oil of these plants is characterized by the presence of p-methoxy-β-cyclopropylstyrene, (*E*)-β-farnesene, β-sesquiphellandrene, and cis-calamenene as major components [63].

In the case of *T. parthenium*, extensive studies have evaluated its major compounds, which include many secondary metabolites, such as camphor, chrysanthenyl acetate, comphene, and bornyl acetate [64,65,66] (Figure 2). 

In addition, the hot water extracts of *T. parthenium* and *T. disciforme* revealed the presence of melatonin in flowers [67]. Another study reported the detection of an acetylene derivative of dioxaspiran in the chloroform extract of *T. disciforme* [68].

The particularly volatile compounds of *T. auriculatum* from Saudi Arabia were characterized as fatty acids and their derivatives [69]. The main compounds of *T. callosum* chloroform extract have been identified as hexadecanoic and linoleic acids [57,63] (Figure 4), while the predominant compound in *T. callosum* is moretenol (11.71%) (Figure 2). Moreover, studies have demonstrated that the most common compounds in flower oil are linoleic acid (16.18%), n-hexadecanoic acid (17.88%) (Figure 4), and n-nonacosane (11.04%).

The major compound in the stem oil of *T*. *callosum* includes 1-tricosene (13.41%) (Figure 4), while the main compounds in the root oil of *T. callosum* are cyclotetracosane (5.88%) and n-hexadecanoic acid (6.18%) [62] (Figure 4).

Ref. [62] mentions that chloroform extracts of *T. callosum* flowers, stems, and roots include *p*-methoxy, β cyclopropylstyrene, and β-farnesene as the main compounds. Additionally, the essential oil of *Tripleurospermum disciforme* is predominantly composed of sesquiterpenes β-farnesene, p-methoxy-β-cyclopropyl styrene, β-sesquiphellandrene and cis-calamenene, ρ-methoxy-humulene oxide, benzene acetaldehyde (Figure 2), and heptadecane [57,59,63,70] (Figure 4).

A total of 21 compounds from *T. disciforme* essential oil were analyzed at different stages, including the flowering stages; and variations in the composition were observed. During the flowering stage, β-farnesene (22.46%) and β-sesquiphellandrene (17.85%) were found to be the major compounds, along with p-methoxy-β-cyclopropylstyrene (16.64%), heptadecane (10.60%) p-methoxy-humulene oxide (6.88%), and benzene acetaldehyde (9.30%) [63] (Figure 2).

The amounts of β-farnesene, β-sesquiphellandrene, p-methoxy-β-cyclopropylstyrene, heptadecane, and benzene acetaldehyde were reported to be highest during the flowering stage and decreased after flowering and seed maturation.

A total of 38 compounds from 89.4% of the total essential oil of *T*. *parviflorum* were identified. The essential oil of *T. parviflorum* was shown to contain significant compounds such as β-farnesene (18.4%), β-sesquiphellandrene (10.1%), carvacrol methyl ether (7.9%), and benzene acetaldehyde (7.2%) [71]. *T. auriculatum* was analyzed for flavonoids and sterols/triterpenes (90%), tannins (78%), volatile oils (60%), and alkaloids and coumarins (50%) [72]. *T. parthenium* was reported to contain a large proportion of oxidized monoterpenes [73]. In the essential oil of *T. disciforme*, the major compounds identified were nonterpenoids, including anisole, *p*-1-cyclohexen-1-yl- (55.95%), modephene (10.00%), camphor (43.43%), and *cis*-β-farnesene (11.94%). Additionally, the amount of caryophyllene (1.66%) was found to be higher than that in *T. parthenium.* [57,59,63,73].

It is worth noting that a study analyzing the chemical composition of *T. disciforme* [55] found a relatively small amount of β-esquiphellandrene (0.22%), which deviated from previous data. Another recent study [74] employed techniques such as NMR, electron impact mass spectroscopy, column chromatography, and thin layer- chromatography (see Figure 2) to identify three triterpenoids—taraxasterol, lupeol, and betulinic acid—in the dichloromethane extract of *T. disciforme*. 

Regarding *T. tenuifolium* and *T. parviflorum*, palmitic acid (C 16:0) and linoleic acid (C 18:2) were identified as the main fatty acids (see Figure 4) [75]. Furthermore, saturated fatty acids (SFAs) were found to be present in higher amounts than monounsaturated fatty acids (MUFAs) and polyunsaturated fatty acids (PUFAs) in both *T. tenuifolium* and *T. Parviflorum*. 

## 4. General Uses, Medicinal Uses, and Pharmacological Studies

The genus Tripleurospermum, belonging to the family Asteraceae, has garnered significant interest in the medical field. Ethnobotanical studies have revealed that *Tripleurospermum* species possess various medicinal properties, including sedative and anti-inflammatory effects; relief of muscle pain, fatigue, and carminative properties [76,77], memory enhancement [78]; cholesterol regulation kidney stone management; treatment of sore throat and wounds healing; respiratory support; diabetes management; cardiac disorders; gastric pain; antiseptic properties and hair care [79,80,81,82,83,84,85,86]. Notably, comprehensive medicinal analyses have demonstrated the therapeutic potential of *T. disciforme*, which exhibits antiulcer effects [87], antibacterial properties [63,88], antioxidant activity [59,68], antimicrobial effects [88], and antispasmodic and antiseptic properties [76,77].

Research on *Tanacetum parthenium*, another member of the Asteraceae family, has shown significant antibacterial activity, potentially attributed to sesquiterpene lactones, such as parthenolide and flavonoids. *T. parthenium* has been used as a neurotonic and antipyretic agent [89], as a relaxant and for muscle ache alleviation, stress management [90] and as a hair dye [91,92]. Additionally, *T. disciforme* extract has demonstrated antimicrobial effects against *S. aureus* and *S. epidermidis* [93,94]. The antioxidant activity of different parts of *T. disciforme* was assessed by evaluating their ability to inhibit linoleic acid peroxidation. Notably, the chloroform extract exhibited a substantial antioxidant effect, slightly lower than that of the standard α-tocopherol [88,95]. Moreover, a study [96]. showed that extracts of *T. parviflorum* and *T. monticola* were effective in treating cough and stomachache and as antipyretic agents. *Tripleurospermum parviflorum* also demonstrated efficacy in the treatment of pharyngeal illnesses and vaginitis [97]. Traditional medicinal practices involve the use of the entire plant of *T. limosum* for the treatment of gastritis [2,96,97]. Additionally, *T. sevanense* has been utilized for hair-care purposes [98].

*Tripleurospermum callosum* flowers have been recommended to treat urinary tract disorders, kidney stones, shortness of breath, common cold, asthma, and bronchitis and as a panacea [70]. Furthermore, *T. auriculatum* was found to be effective in the treatment of neuromuscular blockade and mild hypoglycemia [72]. *T. inodorum* is known for its efficacy in alleviating gastrointestinal pain and its anti-inflammatory properties [99]. Moreover, *T. inodorum* was historically utilized in prehistoric painting techniques, and it is important to mention that apigenin, a compound found in this species, contributes to its yellow coloration [100].

### 4.1. Antioxidant Activity

The antioxidant activity of the essential oils from the newly discovered species *T. insularum*, belonging to the genus *Tripleurospermum* [19], was investigated using 2-diphenyl-1-picrylhydrazyl (DPPH) and ferric-reducing activity (FRAP) assays [59]. The essential oil’s ability to scavenge stable free radicals and reduce metal intermediates may be attributed to the substantial presence of sesquiterpenes, which have known antioxidant properties [59].

The antioxidant activity of *T. insularum* is comparable to that of other *Tripleurospermum* species, such as *T. disciforme* and *T. oreades* [91], which are recognized for their abundance of caffeoyl derivatives, which are regarded as significant compounds for the antioxidant potential of the Asteraceae family [101]. The aqueous extract of *T. oreades* also showed antioxidant activity [91].

In addition, the chloroform and hydroalcoholic extracts of *T*. *disciforme* have been associated with antioxidant and antiulcer properties [68,102]. The antioxidant activity of different fractions of *T. disciforme* was assessed using different methodologies, namely the linoleic peroxidation method, 2-diphenyl-1-picrylhydrazyl (DPPH) assay, Ferric reducing antioxidant power (FRAP) assay, oxygen radical absorbance capacity (ORAC), and cupric ion reducing antioxidant capacity (CUPRAC) assay [68,103] (Table 1). Furthermore, the genus *Tripleurospermum*, specifically *T. disciforme*, showed significant antioxidant activity and elevated levels of total phenolic compounds and flavonoids [90].

It is important to note that the low temperatures at higher altitudes represent a crucial environmental factor contributing to the enhanced biosynthesis of several antioxidants, despite the genetic differences between different species [103,104]. Among the essential oil compounds of *T. inodorum*, matricaria ester exhibited modest antioxidant activity [105]. Recent findings [2] demonstrated the antioxidant activity of *T. limosum*, with notable effectiveness observed in aqueous, methanol, and ethanol extracts.

**Table 1:** Antioxidant activity of the genus *Tripleurospermum*.

Taxon	Part of Plant	Extract	Type of Study
*T. insularum*	Aerial parts, flower, petal, leaf	Essential oil	DPPH (radical scavenging activity), FRAP (ferric-reducing activity) [59]
*T. disciforme*	Peta, flowers, leaf	Water	FRAP assay [91]
*T. oreades*	Aerial parts, flower	Water	DPPH-RS, linoleic acid peroxidation [68,91]
*T*. *disciforme*	Petal, flower, leaf	Water	Linoleic acid peroxidation [68]
*T. limosum*	Leaf, flower, root	Water, methanol, ethanol, acetone, ethyl acetate, ethyl ether, dichloromethane, or hexane	DPPH, ABTS, hydroxyl radical assay, Superoxide radical assay, FRAP, CUPRAC assay, iron chelating assay, copper chelating assay, H_2_O_2_ assay, β-carotene bleaching assay [2], NO assay
*T. inodorum*	Aerial parts	Essential oil Matricaria ester	DPPH [106]
*T. rosellum*, *T. corymbosum*, *T. temskyanum*, *T. caucasicum*, *T. conoclinum*, *T. callosum*, *T. Temskyanum*, *T. oreades*, *T. ziganaense*, *T. corymbosum*, *T. decipiens*, *T. disciforme*, *T. elongatum*, *T. Fissurale*, *T. heterolepis*, *T. hygrophilum*, *T. indorum*, *T. Kotschyi*, *T. melanolepis*, *T. microcephalum*, *T. monticola*, *T. oreades var. oreades*, *T. oreades var tchihatchewii*, *T. parvifiorum*, *T. Pichleri*, *T. rosellum var. album*, *T. rosellum var album*, *T. sevanese*, *T. subnivale*, *T. tempskyanum*, *T. tenuifolium*, *T. transcaucasicum*	Leaf, flower, root	Methanol	ORAC, DPPH, FRAP, CUPRAC [103]

### 4.2. Antimicrobial Activity

Previous studies have investigated the antibacterial properties of Asteraceae plant species and have found a significant inhibitory effect [89,107]. The inhibitory effects of different essential oils have also been examined (Table 2). Most authors have reported a stronger inhibitory effect of essential oils against Gram-positive bacteria than against Gram-negative bacteria [73,92,108,109]. Interestingly, it was found that the levels of flavonoids in Gram-negative bacteria are higher than those in Gram-positive bacteria. 

A noteworthy study explored and elucidated the antiyeast activity of *T. disciforme*, which is attributed to the presence of farnesol [92], a crucial compound in the essential oil of the *Tripleurospermum* genus. Additionally, flavonoids were identified as antimicrobial agents, exhibiting direct antibacterial activity, synergy with antibiotics, and disease elimination in various studies [110]. The methanol extract of the genus *T. disciforme* showed antimicrobial activity against *S*. *aureues* and *S. epidermidis* [88]. In addition, the essential oil of *T. disciforme* showed antimicrobial activity against *Staphylococcus subtilis* and *Bacillus cereus* [63]. The phytochemical composition analysis revealed that compounds such as modephene, *cis*-β-farnesene, β-sesquiphellandrene, anisole, and *p*-1-cyclohexen-1-yl are responsible for the antibacterial activity in the essential oil of T. disciforme. Additionally, these essential oil compounds are present in high concentrations during the flowering period.

Furthermore, the antibacterial activity of essential oils is not solely dependent on their main compounds; minor compounds can also exhibit significant efficacy. Sesquiterpenes, for instance, have demonstrated inhibitory effects. Additionally, a study [111] highlighted the antibacterial activity of flavonoids, specifically kaempferol and quercetin, against *Propionibacterium acnes*.

Apigenin was identified as a compound responsible for suppressing *S. typhi*, *Proteus mirabilis*, and *P. aeruginosa* [112]. Another study investigated the eradication of *S. aureus*, MRSA, and methicillin-sensitive S. aureus using apigenin and luteolin [113]. Researchers provided evidence supporting the therapeutic and disinfectant properties of *T. disciform* in improving acne and resolving skin issues, particularly in teenagers [88]. Moreover, a recent report showed the strong antibacterial activity of *T. parthenium* essential oil against *Aspergillus brasiliensis* [73]. Previous findings demonstrated the strong antibacterial effects of all extracts from *T. Parviflorum*, including ethanol, methanol, and ethyl acetate extracts against *Staphylococcus aureus* ATCC 29213. However, none of the tested extracts showed any effect on *Candida albicans* [114]. Furthermore, another investigation reported the high inhibitory activity of *T. disciforme* essential oil against *Klebsiella pneumoniae*, *Shigella dysenteriae*, *Escherichia coli*, and *Candida albicans* [73].

**Table 2:** Antibacterial, antifungal, and antiparasitic activities of the genus *Tripleurospermum*.

Part of Plant	Type of Study	Extract	Biological Activity
Aerial part (top flower)	Cup plate diffusion method	Methanol	*S*. *aureus* and *S. epidermidis* [89]
Flower	MIC	Essential oil	*Staphylococcus subtilis* and *Bacillues cereus* [63]
Leaf	Disc diffusion assay	n-Hexane, methanol, ethanol, ethyl acetate, and water extract	*Escherichia coli* ATCC 29998, *Escherichia coli* ATCC 25922, *Escherichia coli* ATCC 11230, *Staphylococcus aureus* ATCC 29213, *Staphylococcus aureus* ATCC 6538P, *Enterobacter cloacae* ATCC13047, *Enterococcus faecalis* ATCC 29212, *Pseudomonas aeroginosa* ATCC 27853, and *Candida albicans*, ATCC 10239 [113]
Flower	Agar well diffusion (AD) assay, MIC, MBC /MFC	Essential oil	*Aspergillus brasiliensis*, *Klebsiella* pneumonia, Shigella dysenteriae, *Escherichia coli* [73]

### 4.3. Anti-Inflammatory Activity

*T. disciforme* was shown to have significant anti-inflammatory and analgesic effects by inhibiting the release of prostaglandins and other mediators [115]. Notably, fatty acids have been recognized for their protective properties and diverse biological activities, including anti-inflammatory effects [87,99,108,116].

Previous studies reported the remarkable inhibitory effect of ethyl acetate extracts from *T. tenuifolium and T. parviflorum* on overall in vivo anti-inflammatory activity [117]. Palmitic acid is the major and important component of *T. tenuifolium.* The substantial presence of linoleic acid and palmitic acid accounts for the significant anti-inflammatory activity observed (Table 3).

The anti-inflammatory and analgesic activities of *T. disciforme* were assessed using carrageenan-induced edema, formalin, and the tail-flick test. Extracts of *T. disciforme* demonstrated remarkable anti-inflammatory and analgesic properties in the tested models [118]. An extract of *T. disciforme* was shown to be nontoxic at analgesic doses. Furthermore, the administration of large amounts of *T. disciforme* further confirmed its potent anti-inflammatory effects [118]. 

Studies on the hydroalcoholic extracts of *T. disciforme* have revealed its remarkable ability to protect against ulcer formation in rats with pyloric ligation, indicating the involvement of additional mechanism(s) beyond its acid-reducing activity. Additionally, high doses of *T. disciforme* exhibited effectiveness upon administration via injection [87]. The intensity of ulceration is typically assessed by parameters such as ulcer score or ulcer incidence [117,119]. Flavonoids and essential oils containing important secondary metabolites in the flowers of *T. disciform* were identified as contributors to the anti-inflammatory activity of the genus *T. disciform* [120]. Furthermore, a study [121] identified the major flavonoid compounds of *T. disciform*, including apigenin, apigenin-7-glucoside, apigenin-7-glucuronide, luteolin, luteolin-7-glucoside, luteolin-7-glucuronide, quercetin, quercetin-7-glucoside, and chrysoeriol, which demonstrated effective anti-inflammatory activity [122].

**Table 3:** Anti-inflammatory activities of the genus *Tripleurospermum*.

Part of Plant	Identified Compounds	Extract	Biological Activity
Aerial part	Linoleic and palmitic acids	n-Hexane Aqueous, ethyl acetate, and methanol	Anti-inflammatory, by carrageenan- and serotonin-induced paw edema acetic-acid-induced increase in capillary permeability models [114]
Flower	Flavonoids such as apigenin, quercetin, patuletin, luteolin, and their glucosides	Water	Analgesic and anti-inflammatory, evaluated by formalin test, by inhibiting the cyclooxygenase(COX)-mediated conversion of arachidonic acid to prostanoids [118]
Flower	Flavonoids and essential oil compounds [122]	Hydroalcoholic and chloroform	Antiulcerogenic potential [68,103]; anti-inflammatory, by carrageenan induced edema, formalin, and tail-flick test [114]
Flower	Flavonoids such as flavones and flavonols, tannins, and essential oil	Hydroalcoholic	Protection against gastric ulcers in pylorus-ligated rats [87]

### 4.4. Cytotoxic Activity

The cytotoxic activity of the methanol extract of *T. disciforme* was investigated against several human cancer cell lines, including human lung adenocarcinoma (A549), human breast adenocarcinoma (MCF7), hepatocellular carcinoma (HepG2), and human colon carcinoma (HT-29) [123] (Table 4).

The cytotoxic activity of various extracts of *T*. *parviflorum*, namely methanol, n-hexane, ethanol, ethyl acetate, and water extracts, were assessed against brine shrimp (LC50 < 1000) [113]. Among the tested extracts, the methanol extract showed the highest cytotoxic activity compared with that of the other extracts tested. All extracts, except the aqueous one, exhibited higher activity than the cytotoxic agent umbelliferone. However, the cytotoxicity of the *T. parviflorum* extracts was lower than that of colchicine.

Moreover, the dichloromethane extract of *T. disciforme* demonstrated antitumor activity against human gastric carcinoma (AGS) and mouse skin fibro sarcoma (WEHI-164) cell lines [74]. However, *T. disciforme* did not exhibit cytotoxic effects on Pc12 cells [124]. Notably, the essential oil of the genus *T. inodorum* contains a compound called matricaria ester, known for its potent cytotoxicity against *Artemia salina.* [105].

Recent findings revealed that the methanol extract of *T. limosum* exhibited low cytotoxicity at the lowest concentration tested and no cytotoxicity at the other three concentrations [2].

## 5. Conclusions

The primary group of phytochemicals found in the essential oils of the genus *Tripleurospermum* exhibit significant potential as sources of various compounds, such as cis-β-farnesene, 1-tricosene, phytol, guaia-6,10(14)-dien-4β-ol, γ-cadinol, nonacosane, decanoic acid, caryophyllene oxide, linolenic acid, palmitoleic acid, heptadecane, n-hexadecanoic, n-nonacosane, apigenin, apigenin-7-glucoside, apigenin-7-glucuronide, luteolin, luteolin7-glucoside, luteolin-7-glucuronide, quercetin, quercetin-7-glucoside, melatonin, and chrysoerio. These compounds have demonstrated diverse therapeutic applications in multiple fields.

Traditionally, these plants have been commonly employed for the treatment of gastrointestinal disorders, inflammation, throat ailments, vaginitis, dysentery, fever, skin diseases, urinary tract disorders, kidney stones, shortness of breath, common cold, asthma, bronchitis, and muscular pain, and as a sedative, a gastro tonic, an antihemorrhagic, a carminative, a relaxant, stress-relieving agents, hair colorants, and as panaceas. This review provided an overview of the medicinal uses of *Tripleurospermum* species across different countries, thus highlighting their remarkable potential for various biological activities. Notwithstanding, further future research is required to identify the individual bioactive compounds present in *Tripleurospermum* species and elucidate their mechanisms of action.

## Figures and Tables

**Table 4:** Cytotoxic activities of the genus *Tripleurospermum*.

Taxon	Activity	Extract	Biological Activity
*T. disciforme*	(−)	Methanol	(A549), (MCF7), (HepG2), and (HT-29) [123]
*T. parviflorum*		n-Hexane	
	(+)	methanol	Brine shrimp [113]
		ethanol, ethyl acetate, and water	
*T. disciforme*	(+)	Dichloromethane	(AGS) and (WEHI-164) [74]
*T. disciforme*	(−)	Methanol	PC12 cells [124]
*T. inodorum*	(+)	Essential oil (matricaria ester	Brine shrimp [105] (*Artemia salina*)
*T. limosum*	(+)	Methanol	TM3 cells [2]

(−), any cytotoxicity activity; (+), cytotoxicity activity.
